# Anti-cancer efficacy of biotinylated chitosan nanoparticles in liver cancer

**DOI:** 10.18632/oncotarget.19146

**Published:** 2017-07-10

**Authors:** Mingrong Cheng, Weiping Zhu, Qing Li, Dejian Dai, Yiming Hou

**Affiliations:** ^1^ Department of General Surgery, Pudong New Area District Zhoupu Hospital, Shanghai 201318, China; ^2^ Department of General Surgery, Shanghai Tianyou Hospital, Tongji University, Shanghai 200000, China; ^3^ Department of Hepatic Surgery, Fudan University Shanghai Cancer Center, Shanghai Medical College, Fudan University, Shanghai 200032, China; ^4^ Department of Medical Clinic, Pujiang Community Health Service Center, Shanghai 201112, China; ^5^ Department of General Surgery, Shanghai Fifth People's Hospital, Fudan University, Shanghai 200240, China

**Keywords:** biotin chitosan, curative effect, drug delivery system, liver cancer, nanoparticles

## Abstract

The present study investigated the synthesis of biotinylated chitosan (Bio-CS) from chitosan using a nanomaterial skeleton with biotin and the successful targeting of the formulation in liver cancer cells. Bio-CS was validated by fourier transformed infrared spectroscopy and hydrogen^-1^ nuclear magnetic resonance spectroscopy. Bio-CS and plasmid DNA were used to construct Bio-CS/plasmid DNA nanoparticles according to the optimal molar ratio of 1:1 and the optimal pH-value of 5.5. Under these conditions, the parameters mean particle size, potential, encapsulation rate and drug loading, were 82.9 nm, +21.8 mV, 85.7% and 35.4%, respectively. Bio-CS exhibited an apparent liver cancer targeting effect *in vitro* and *in vivo*, as demonstrated by confocal laser scanning, green fluorescent protein transfection, and *in vivo* imaging assays. In addition, the Bio-CS/plasmid DNA nanoparticles significantly increased the survival period of the orthotropic liver cancer mouse model compared with the plasmid DNA, with no apparent side effects on the cells. Bio-CS nanomaterials stimulated an immune response in hepatoma cells via increased expression of GM-CSF, IL-21 and Rae-1 markers. The data suggest that Bio-CS increased the inhibition of liver cancer cell proliferation *in vitro* and the activation of the cellular immunity *in vivo*.

## INTRODUCTION

Liver cancer can be therapeutically targeted by enhanced immune surveillance, inhibition of immune escape, activation of the body's innate immunity and the elevated expression of specific liver cancer markers that can be recognized by the immune system. Liver cancer cells employ multiple mechanisms in order to escape recognition of the host immunity and to promote their survival and proliferative capacities [1]. However, immune escape can be avoided by the following processes: Host immunity can be stimulated, notably in the context of anti-tumor cytotoxic T lymphocytes (CTL) and natural killer (NK) cells, whereas the expression of antibodies and/or ligands that are recognized by host immunity in cancer cells may be increased [2]. An example of the latter process includes the natural killer group 2D (NKG2D). The recombinant plasmids of granulocyte-macrophage colony stimulating factor (GM-CSF) and interleukin-21 (IL-21) were synthesized in a previous study [3]. The orthotopic liver cancer model in mice was established by treatment with recombinant plasmids that could activate the immune system, stimulate the activity of CTL and NK cells, and exert significant cytotoxic activity in hepatoma cells. The retinoic acid early transcription factor-1 (Rae-1) is one of the most frequently studied mouse NKG2D ligands [4]. The expression of Rae-1 is induced by NKG2D and is mediated by the DAP10 pathway (the “self-induction hypothesis”) [5]. Cancer cells that express Rae-1 are readily identified by immune cells that facilitate their removal, based on the expression of the Rae-1 protein [6]. Previous studies conducted by our group, demonstrated the construction of pGM-CSF-GFP-IRES-Rae-1-IL-21 as an “immune escape system”. The latter process can improve the activity of NK and CTL cells, reduce the percentage of regulatory T cells, increase the expression of NKG2D ligand Rae-1 in hepatocellular carcinoma cells and promote the recognition of hepatocellular carcinoma cells by CTL and NK cells in order to ultimately enhance immune surveillance and inhibit immune escape [7]. The efficacy of the “immune escape system” in the mouse model of orthotopic liver cancer remains unknown. Thus, the safe and systematic transport of this system to the liver cancer cells warrants further investigation.

Gene therapy usually includes two key factors namely, the contribution of the genes encoding for proteins that increase the treatment efficacy, and the gene delivery system that can effectively transfer genetic material to specific sites [8]. The naked genes are readily degradable by enzymes and have the defects of high renal clearance rate that results in poor cellular uptake and low efficiency of endosomal escape. Consequently, the success of gene therapy depends largely on the development of a safe and effective gene delivery system [9]. Galactosylated chitosan was synthesized from chitosan and galactose in the previous study conducted by our group, and the gene was successfully transfected in hepatocarcinoma tissue [10, 11]. In addition to the cancer cells, this formulation exhibits similar targeting efficacy for normal liver cells. Thus, it is expected that the targeting material used for liver cancer cells is grafted with chitosan to form an effective gene carrier for liver cancer cells. Biotin, also named vitamin H, is a small molecule of approximate molecular weight of 244 Da that requires a specific receptor to exert its biological functions. The biotin receptor can be found on almost all cell surfaces of the human body [12]. Tumor cells require a multitude of nutrients due to excessive and rapid growth, and the biotin receptor is frequently overexpressed in certain cancers that in turn lead to a high binding capacity for the compound compared with the normal cells [13]. It has been reported that the adsorption of biotin in hepatoma cells is 39.6 times higher compared with that in normal hepatocytes [14, 15]. Moreover, the adsorption of biotin in hepatic cancer is increased as the biotin content increases [14, 15]. In the present study, the ligands of biotin (liver cancer targeting) were grafted on chitosan, and Bio-CS nanomaterials with liver cancer targeting effects were synthesized. Bio-CS was prepared as a vector for the delivery of the previously reported plasmid DNA (pGM-CSF-GFP-IRES-Rae-1-IL-21). The targeting and anti-cancer characteristics of Bio-CS / plasmid DNA on liver cancer were explored *in vitro* and *in vivo*. In addition, the immune mechanism was investigated in the mouse orthotopic liver cancer model.

## RESULTS

### Fourier transformed infrared spectroscopy and ^1^H-nuclear magnetic resonance of biotinylated chitosan

**Figure 1:**
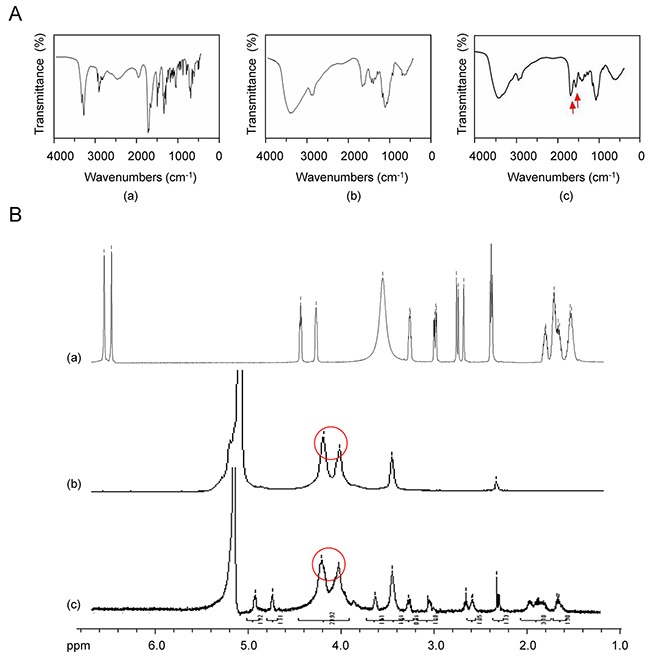
FT-IR and ^1^H-NMR of Bio-CS **(A)** The mass spectrum of Bio-CS: (a) IR spectra of biotin; (b) IR spectra of CS; (c) IR spectra of Bio-CS, red arrow indicates the stretch vibration absorption peak of the newly generated amide bond. **(B)** ^1^H-NMR of Bio-CS: (a) ^1^H-NMR of biotin; (b) ^1^H-NMR of CS, red circle indicates the hydroxyl proton signal peaks; (c) ^1^H-NMR of Bio-CS, red circle depicts the newly generated displacement peak of amide hydrogen. FT-IR, fourier transformed infrared spectroscopy;^1^H-NMR, Hydrogen-1 nuclear magnetic resonance; Bio-CS, biotinylated chitosan; IR, infrared; CS, chitosan.

Nanomaterials, such as biotin, CS, and Bio-CS were determined using IR spectra, and the results of the IR spectra of (a) biotin, (b) CS, and (c) Bio-CS are presented in Figure 1A. The peak at 1,700 cm^–1^ corresponded to the stretch vibration absorption peak of the C=O in the carboxyl group, whereas the peaks at 1,640 and 1,480 cm^–1^ corresponded to the stretch vibration absorption of carboxylic acid and the bending vibration absorption of the N–H group, respectively (Figure 1A(a)). A total of three characteristic amide absorption peaks are indicated in the IR spectra namely, an amide band I at 1,653 cm^–1^, an amide band II at 1,600 cm^–1^, and an amide band III at 1,322 cm^–1^ (Figure 1A(b)). The waves at 3,420 and 2,882 cm^–1^ demonstrated the corresponding peaks for the stretch vibration absorption of O–H and N–H and for C–H, respectively. The IR spectra of biotinylated chitosan exhibited an increase in the intensity of the amide band I (Figure 1A(c)). The peaks at 1,681 cm^–1^, 1,557 cm^−1^ and 1,461 cm^–1^ corresponded to the stretch vibration absorption of the newly generated amide bond, whereas the peak at 1,480 cm^–1^ represented the bending vibration absorption of N–H. These changes were attributed to the formation of the amide bond between the amine of the chitosan and the carboxyl group of biotin that promoted a successful graft of biotin on the chitosan. The characteristic absorption peaks at 2.0, 3.16, and 3.88 parts per million were observed for CS, which were attributed to the protons in the N–CH_3_, amino, and hydroxyl groups, respectively (Figure 1B(b)). A novel double absorption peak between 3.71 and 3.88 parts per million was observed in Bio-CS. The peak in the BIO-CS spectra at 3.4-3.5 parts per million is considered the characteristic peak corresponding to the proton in the biotin molecular structure (Figure 1B(c) and 1B(a). This peak occurred due to the generation of a new amide bond on the displacement peak of hydrogen. The data indicated the successful linkage of biotin to the amino group of CS. Bio-CS nano-material was synthesized with 200 mg biotin, utilizing a biotin content of 1.25 ± 0.25 μmoL/100 mg Bio-CS nano-material. The degree of biotin substitution was 35%.

### Synthesis of Bio-CS/plasmid DNA nanoparticles and their release characteristics *in vitro*

**Figure 2:**
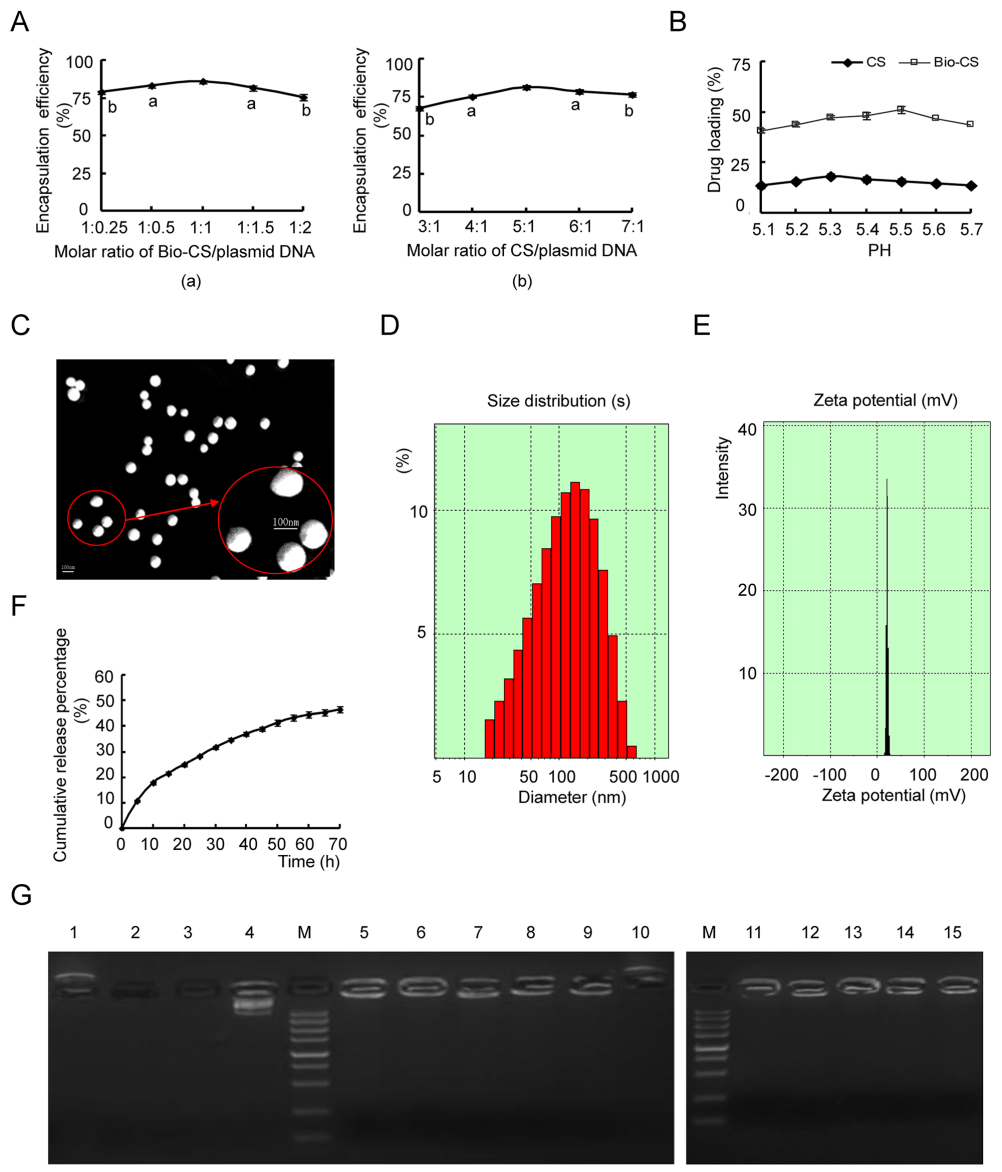
Characteristics of Bio-CS nanoparticles *in vitro* **(A)** The optimal ratio of nanomaterials/ plasmid DNA: (a) Screening of Bio-CS/plasmid DNA for the optimal ratio. Data were analyzed by *t* test. ^a^*P*<0.05, ^b^*P*<0.01, as compared with 1:1 group. (b) Screening of CS/plasmid DNA for the optimal ratio. Data were analyzed by *t* test. ^a^*P*<0.05, ^b^*P*<0.01, compared with the 1:1 group. **(B)** Impact of different pH value on drug loading of Bio-CS and CS nanoparticles. **(C)** Transmission electron micrograph of Bio-CS/plasmid DNA nanoparticles. Scale bar=100nm. **(D)** The particle size graph of Bio-CS/plasmid DNA nanoparticles (n=3). **(E)** The zeta potential graph of Bio-CS/plasmid DNA nanoparticles (n=3). **(F)** The *in vitro* release curve of nanoparticles in simulated body fluid (37°C, pH 7.4). **(G)** Electropherogram of Bio-CS/plasmid DNA nanoparticles with DNase I and fetal bovine serum. (Lane 1, Bio-CS; Lane 2, plasmid DNA + plasma digested at 37°C for 1 h; Lane 3, plasmid DNA + DNase I digested at 37 °C for 1 h; Lane 4, plasmid DNA; M, Marker 5,000 (5,000, 3,000, 1,500, 1,000, 750, 500, 250, 100, and 50 bp ordered from top to bottom); Lane 5, Bio-CS/plasmid DNA; Lane 6, Bio-CS/plasmid DNA+ DNase I digested at 37 °C for 30 min; Lane 7, Bio-CS/ plasmid DNA+ DNase I digested at 37°C for 1 h; Lane 8, Bio-CS/ plasmid DNA+ DNase I digested at 37 °C for 2 h; Lane 9, Bio-CS/ plasmid DNA+ DNase I digested at 37 °C for 8 h; Lane 10, Bio-CS + DNase I digested at 37 °C for 8 h; Lane 11, Bio-CS/ plasmid DNA stored at 4 °C for 1 month; Lane 12, Bio-CS/ plasmid DNA stored at 4 °C for 2 months; Lane 13, Bio-CS/ plasmid DNA stored at 4 °C for 3 months; Lane 14, Bio-CS/ plasmid DNA stored at 4 °C for 4 months; Lane 15, Bio-CS/ plasmid DNA stored at 4 °C for 5 months. Bio-CS, biotinylated chitosan; CS, chitosan.

The encapsulation efficiencies of the Bio-Cs/plasmid DNA and CS/plasmid DNA increased gradually to a maximum value and then decreased to levels similar to those noted at the highest (1:0.25) and lowest (3:1) molar ratios, respectively (Figure 2A(a-b)). The maximum efficiency was noted at the molar ratio of 1:1 for the Bio-Cs/plasmid DNA and at the molar ratio of 5:1 for Cs/plasmid DNA (Figure 2A(a-b)) The drug loading of the CS/plasmid DNA increased gradually during an increase in the pH value from 5.1 to 5.3 (Figure 2B). Concomitantly, the drug loading decreased as the pH-value increased from 5.3 to 5.7 (Figure 2B). The optimal pH-value of the drug loading was 5.3 and 5.5 for the CS and Bio-CS formulations, respectively. Electron microscopy demonstrated that the Bio-CS/plasmid DNA particles exhibited regular spherical shape, with a smooth surface, a uniform size, and no adhesion between nanoparticles (Figure 2C). The parameters, radius of the nanoparticles, zeta potential, encapsulation efficiency and drug loading were 82.9±5.6 nm, +21.8±1.3 mV, 85.7% and 35.4%, respectively (Figure 2D and 2E). The release profile in Bio-CS/plasmid DNA nanoparticles occurred in two stages namely, the rapid-release and the slow-release stage (Figure 2F). The rapid-release stage was observed from 0 to 15 h. The cumulative release percentage was 26.3% in this stage, which may be due to the diffusion of the plasmid DNA in the solution. The slow-release stage occurred between 15 h and 70 h. A total of 24.9% of plasmid DNA was released in this stage due to the degradation of the Bio-CS materials.

### Gene protection of Bio-CS materials

Gel electrophoresis of plasmid DNA indicated a single band in the absence of digestion, while Bio-CS/plasmid DNA nanoparticles, which were digested with DNase *I* at 37 °C for 30 min to 8 h, revealed additional bands (Figure 2G). The Bio-CS/plasmid DNA nanoparticles digested with plasma at 37°C for 8 h, exhibited an apparent band, while the bands of the plasmid DNA that were digested with plasma and/or DNase *I* at 37°C for 30 min disappeared. The Bio-CS nanoparticles in the presence and/or absence of DNase I yielded positively charged bands. Taken collectively, the data suggested that Bio-CS materials were positively charged and could not be digested by the enzymes. No leakage of plasmid DNA was observed in the gel wells, indicating that the Bio-CS/plasmid DNA stored at 4°C retained its stability during the time period of 1 to 5 months.

### Bio-CS nanoparticle-mediated targeting of liver cancer cells *in vitro* and *in vivo*

**Figure 3:**
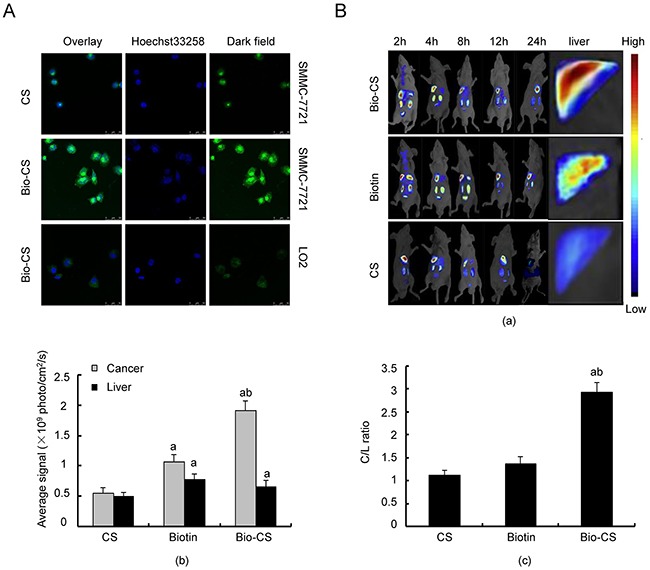
Bio-CS nanoparticles hepatoma cell targeting *in vitro* and *in vivo* **(A)** Bio-CS nanoparticles hepatoma cell targeting *in vitro*. SMMC-7721 and LO2 cells were incubated with fluorescein isothiocyanate (FITC)-labeled GA-CTS for 4 h (*n* = 3). Fluorescence images were screened by confocal microscopy following nuclear staining with the fluorescent dye Hoechst 33258. **(B)** Dynamic image of Bio-GS nanoparticles in orthotopic transplantation liver cancer model. Following establishment of the orthotopic liver transplantation model at day 5, RBITC-CS, RBITC-Biotin and RBITC-Bio-CS nanoparticles were labeled by isothiocyanate Luo Danming B and were injected via the tail vein. The dynamic distributions in the mouse body were observed by an *in vivo* imaging system at 2, 4, 8, 12 and 24 h (n=3). (a): Image of orthotopic transplantation liver cancer model by the *in vivo* imaging system for each group at the different time points. (b): Histogram of the fluorescence photon number in liver cancer and liver tissues at the time point of 24 h. The data were analyzed by ANOVA test. ^a^*P*<0.01, compared with CS; ^b^*P*<0.01, compared with Biotin. (c): Histogram of C/L ratio in the liver at the time point of 24 h. The data were analyzed by ANOVA test. ^a^*P*<0.01, compared with CS; ^b^*P*<0.01, compared with Biotin. Bio-CS, biotinylated chitosan; SMMC-7721, human hepatocellular carcinoma cells; LO2, normal liver cells; FITC, fluorothioisocyanate; RBITC, Rhodamine B isothiocyanate; ANOVA, analysis of variance.

The endocytosis processes of Bio-CS nanoparticles in SMMC-7721 and LO2 cells were analyzed using confocal microscopy. The fluorescence intensity was used as a marker of monitoring endocytosis *in vitro* (Figure 3A). The endocytosis of Bio-CS nanoparticles in SMMC-7721 cells exhibited the greatest potency, as determined by the detection of green fluorescence, which was significantly higher compared with that of CS. Furthermore, the green fluorescence noted in LO2 cells following the treatment of Bio-CS nanoparticles, was lower compared with that noted in SMMC-7721 cells.

Nanoparticles were labeled using Rhodamine B isothiocyanate in order to examine their dynamic distribution in the orthotopic transplantation liver cancer model. The fluorescence intensity in CS was considerably low in normal liver and/or liver cancer tissue at 24 h, while it was significantly enhanced in the presence of biotin (Figure 3A and 3B, P < 0.01). The highest fluorescence intensity in liver cancer cells was observed in Bio-GS compared with that noted in the other groups (*P* < 0.01), whereas the fluorescence intensity in the liver tissue significantly increased in Bio-GS compared with the CS and biotin groups (*P* < 0.01).

The C/L ratio reflected the targeting of the nanoparticels to the liver cancer cells. Bio-CS exhibited the highest C/L ratio (Figure 3C, *P* < 0.01), which indicated that the Bio-CS in the presence of ligands exhibited the greatest targeting effect on the liver cancer cells (*P* < 0.01). Although the fluorescence intensity of normal liver and liver cancer tissues in the presence of biotin was significantly increased compared with CS, the C/L ratio was not significantly different between biotin and CS. This suggested that biotin was more efficient in targeting the liver tissues, following the synthesis of Bio-CS in the presence of biotin and CS.

### Cell transfection of Bio-CS nanoparticles in liver cancer tissues

**Figure 4:**
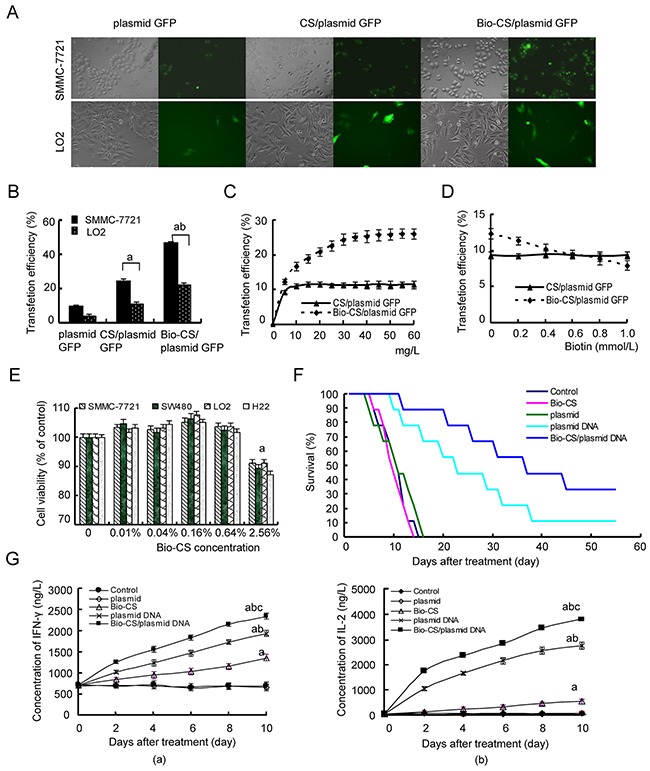
Bio-CS nanoparticles induce cytotoxicity in liver cancer cells *in vitro* and *in vivo* **(A)** Fluorescence images of cells transfected with Bio-CS nanomaterials. SMMC-7721 and LO2 cells were transfected with Bio-CS/plasmid GFP and CS/plasmid GFP nanoparticles and incubated for 48 h. The green fluorescence was observed by a fluorescence microscope. **(B)** Transfection efficiency of cells was detected by flow cytometry. The data were analyzed by ANOVA test. ^a^*P*<0.01, compared with plasmid GFP; ^b^*P*<0.01, compared with CS/plasmid GFP. **(C)** Cellular uptake of Bio-CS/plasmid GFP nanoparticles. The final concentrations of Bio-CS/plasmid GFP and CS/plasmid GFP nanoparticles were 5, 10, 15, 20, 25, 30, 35, 40, 45, 50, 55, and 60 mg/L, respectively; they were subsequently incubated with SMMC-7721 cells for 48 h, and the transfection efficiency was detected using flow cytometry. The data between each point time were analyzed by the *t* test. **(D)** The inhibitory effect of biotin on Bio-CS/plasmid GFP nanoparticles. The concentrations of biotin in the culture medium were 0, 0.2, 0.4, 0.6, 0.8, and 1.0 mmol/L. The transfection rates of Bio-CS/plasmid GFP and CS/plasmid GFP nanoparticles on SMMC-7721 were observed. **(E)** The observation of cytotoxicity of Bio-CS materials. SMMC-7721, SW480, and LO2 cells were cultured with Bio-CS nanomaterials at the concentration range of 0 to 2.56% for 3 days, and the effects of the Bio-CS materials on cell proliferation were evaluated using CCK-8. ^a^*P*<0.01, compared with 0%. **(F)** The inhibitory effect of Bio-CS/plasmid DNA nanoparticles on an orthotopic liver cancer model in mice. On day 5 following establishment of the model, the mice were treated with Bio-CS /plasmid DNA, CS/plasmid DNA, plasmid DNA, Bio-CS, and phosphate buffered solution (PBS), and the survival of each group was observed (*n* = 9). The survival times of the mice in each group were analyzed. ^a^*P*<0.01, compared with control;^b^*P*<0.05, compared with CS/ Bio-CS /plasmid DNA. **(G)** Serum levels of INF-γ and IL-2 were detected by ELISA. The blood was collected in order to obtain serum samples for the detection of INF-γ and IL-2 levels following treatment for the periods of 0, 2,4,6,8 and 10 days. The data are represented as mean±SD. (a): The serum levels of INF-γ in each group; (b): The serum levels of IL-2 in each group. The data were analyzed by ANOVA test. ^a^*P*<0.01, compared with control and plasmid; ^b^*P*<0.01, compared with Bio-CS, ^c^*P*<0.01, compared with plasmid DNA. Bio-CS, biotinylated chitosan; CS, chitosan; SMMC-7721, human hepatocellular carcinoma cells; LO2, normal liver cells; GFP, green fluorescent protein; CCK-8, Cell Counting Kit-8; PBS, phosphate buffered saline; INF-γ, Interferon-γ; IL-2, Interleukin-2;ELISA, enzyme linked immunosorbent assay; ANOVA, analysis of variance.

The intensity of the green fluorescence of SMMC-7721 cells that were transfected with Bio-CS/plasmid-GFP was higher compared with that noted for the CS plasmid-GFP (Figure 4A and 4B). The transfection efficiency of the aforementioned plasmids in LO2 cells was higher than that for the CS and Bio-CS plasmids in SMMC-7721 cells. The data further confirmed that Bio-CS nanoparticles exhibited a potent targeting effect on liver cancer cells *in vitro*. The transfection efficiency in SMMC-7721 cells that were treated with different concentrations of Bio-CS/plasmid-GFP nanoparticles, was significantly higher compared with that noted in LO2 cells with the corresponding concentrations of CS/plasmid-GFP nanoparticles (Figure 4C). This indicated that the amount of Bio-CS uptake in SMMC-7721 cells was significantly higher compared with that noted for the CS nanoparticles (*P* < 0.01). The present study further indicated that the uptake of Bio-CS in SMMC-7721 cells reached a saturation effect. At concentrations higher than 30 mg/L, the uptake of Bio-CS in SMMC-7721 cells did not increase significantly (*P* > 0.05). The transfection rate of Bio-CS/plasmid GFP nanoparticles in SMMC-7721 cells decreased, following an increase in the concentration of biotin in the culture solution (Figure 4D, *P* < 0.01). The transfection rate of Bio-CS/plasmid GFP nanoparticles in SMMC-7721 cells was lower than that of the CS/plasmid GFP nanoparticles (*P* < 0.01) at a biotin concentration higher than 0.6 mM, which suggested that the uptake of Bio-CS in SMMC-7721 cells was competitively inhibited by biotin.

### Cytotoxic effects of Bio-CS nanoparticles

In order to examine the potential cytotoxicity of Bio-CS nanoparticles, SMMC-7721, SW480, H22, and LO2 cells were cultured with different concentrations of Bio-CS nanoparticles for 72 h. The treatment of the cells with Bio-CS at a concentration range of 0.01% to 0.64% of nanoparticles, exhibited no inhibition of cellular proliferation (Figure 4E). The proliferation rates of SMMC-7721, SW480, H22, and LO2 cells were higher than 100% (Figure 4E). No significant differences were observed between the aforementioned treatment of Bio-CS and the control group, which suggested that the high concentrations of Bio-CS exhibited no apparent cytotoxic effect on the cells. The concentration of Bio-CS in this gene targeting delivery system was 0.04% that was consistent with the absence of cytotoxic side effects.

### Bio-CS/plasmid DNA nanoparticles increased *in vivo* survival in the orthotopic liver transplantation model

Following the establishment of the orthotopic liver transplantation model, the mice were randomly assigned into 6 groups and treated as described previously. The survival analysis was carried out using Kaplan–Meier curves (Figure 4F). The mice of the control, Bio-CS, plasmid, and plasmid DNA groups exhibited reduced survival from days 6, 6, 5 and 7, following treatment, and all animals did not survive by days 14, 14, 16 and 20, respectively. The median survival time periods of the aforementioned groups were 11, 10, 11 and 11 days, respectively. The mice of the CS/plasmid DNA and Bio-CS/plasmid DNA groups exhibited reduced survival from day 10 and 12, respectively following treatment, and only 1 and 2 mice survived by day 60, respectively. The median survival time periods of the aforementioned groups were 14 and 37 days, respectively. The median survival time in the Bio-CS/plasmid DNA group was the highest, and the median survival times in the control, Bio-CS and plasmid groups were the lowest (*P* < 0.01). The results indicated that nanoparticles could increase the efficacy of drug administration and prolong the survival time of the mice.

### Bio-CS/plasmid DNA nanoparticles increase the expression of INF-γ and IL-2 in the orthotopic liver transplantation model

The levels of IFN-γ and IL-2 increased significantly at the time points of 2, 4, 6, 8 and 10 days in the Bio-CS, plasmid DNA and Bio-CS/plasmid DNA groups compared with those noted at the baseline (day 0) (Figure 4G, *P* < 0.01). No significant differences in the levels of IFN-γ and IL-2 were noted among the time points investigated in the control and plasmid groups (*P* > 0.05).

### mRNA and protein expression of GM-CSF, IL-21 and Rae-1 levels in H22 cells

**Figure 5:**
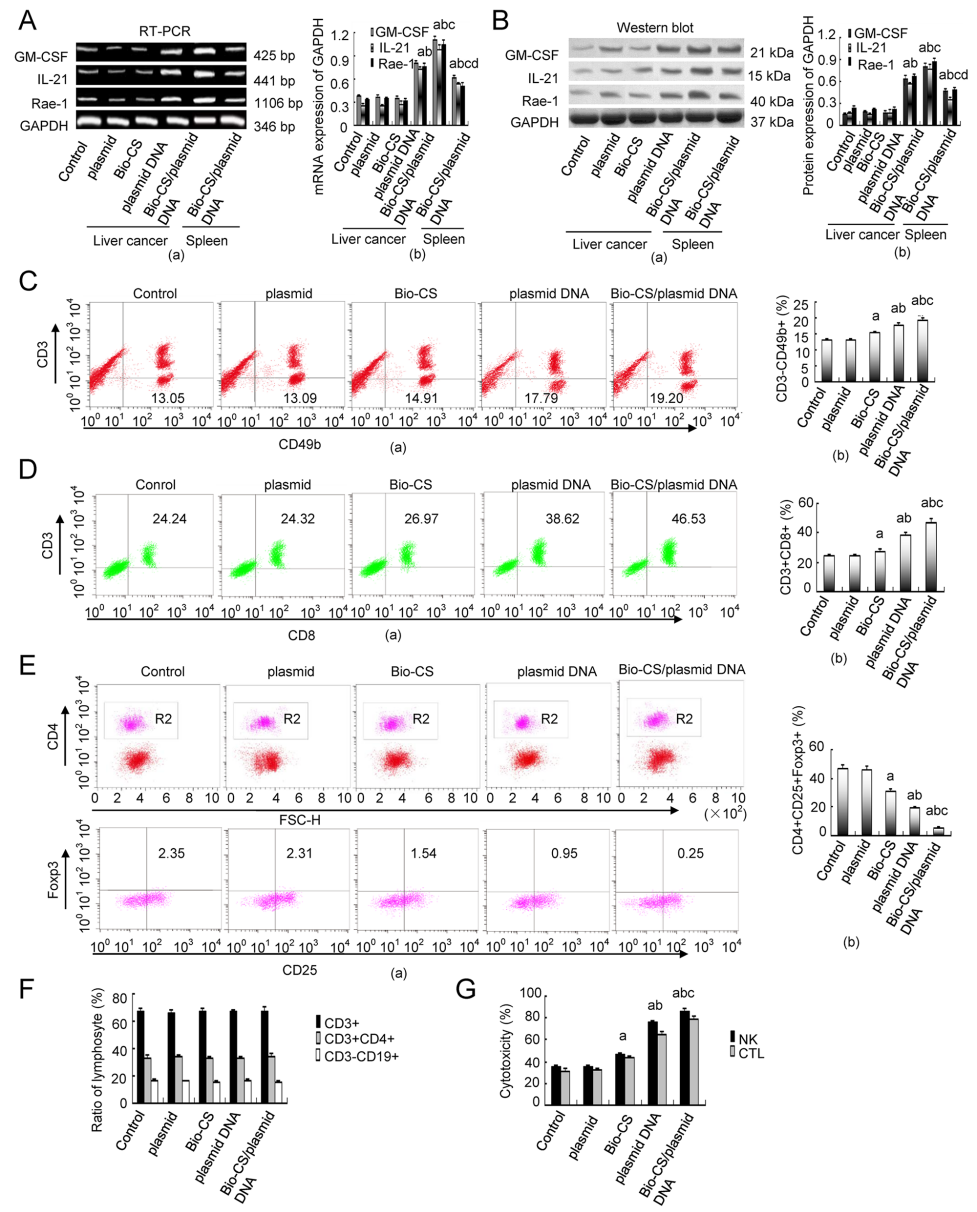
Bio-CS/plasmid DNA nanoparticles increase cellular immunity in mice with orthotopic liver cancer At 10 d following treatment, the mice were sacrificed and the tumor tissue and spleen tissue that was extracted was assessed by RT-PCR and Western blot analysis in order to determine GM-CSF, IL-21 and Rae-1 expression. Flow cytometry was employed to determine the splenic total T cells (CD3^+^), Th cells (CD3^+^CD4^+^), CTL (CD3^+^CD8^+^), B cells (CD3^-^CD19^+^), NK cells (CD3^-^CD49b^+^) and the proportion of Treg cells (CD4^+^CD25^+^Foxp3^+^). The data were analyzed by ANOVA test. ^a^*P*<0.01, compared with control and plasmid;^b^*P*<0.01, compared with Bio-CS,^c^*P*<0.01, compared with plasmid DNA;^d^*P*<0.01, compared with Bio-CS/plasmid DNA. **(A)** RT-PCR analysis of GM-CSF, IL-21 and Rae-1 in H22 cells transfected with Bio-CS / plasmid DNA nanoparticles *in vitro*. (a): Histograms of GM-CSF, IL-21 and Rae-1 expression; (b): Electrophoregrams of GM-CSF, IL-21 and Rae-1. **(B)** Western blot analysis of GM-CSF, IL-21 and Rae-1 in H22 cells transfected with different plasmids *in vitro*.(a): Histograms of GM-CSF, IL-21 and Rae-1 expression; (b): Electrophoregrams of GM-CSF, IL-21 and Rae-1. **(C)** Flow chart and frequency of NK cells in a mixed population of spleen cells. (a): Histograms of NK frequency; (b): flow chart of NK. **(D)** Flow chart and frequency of CTLs in a mixed population of spleen cells. (a): Histograms of CTLs frequency; (b): flow chart of CTLs. **(E)** Flow chart and frequency of Treg cells in a mixed population of spleen cells. (a): Histograms of Treg cells frequency; (b): flow chart of Treg cells. **(F)** Ratios of T cells (CD3^+^), Th cells (CD3^+^CD4^+^), and B cells (CD3^-^CD19^+^) in the spleen following 10 d of treatment. **(G)** NK and CTL-mediated cytotoxity as determined by MTT assays following 10 d of treatment. The data were expressed as mean ± standard deviation (n=3). Bio-CS, biotinylated chitosan; CS, chitosan; Th, helper T; CTL, cytotoxic lymphocyte; NK, natural killer cell; Treg, regulatory cells; ANOVA, analysis of variance; GM-CSF, granulocyte macrophage-colony stimulating factor; IL-21, interleukin-21; Rae-1, retinoic acid early transcription factor-1;MTT, methylthiazolyldiphenyl-tetrazolium bromide.

Following 10 days of treatment, the mRNA and protein expression levels of the markers GM-CSF, IL-21 and Rae-1 increased in the Bio-CS group compared with those in the Bio-CS/plasmid DNA and plasmid DNA groups (*P* < 0.01) as shown in Figure 5A(a-b) and 5B(a-b). The mRNA and protein expression levels of these parameters in the Bio-CS/plasmid DNA group were higher compared with those noted in the plasmid DNA group (*P* < 0.01), although no significant differences were observed among Bio-CS, plasmid and control groups (*P* > 0.05). The mRNA and protein expression levels of GM-CSF, IL-21 and Rae-1 in spleen tissues decreased compared with the corresponding expression in liver cancer tissue in the Bio-CS/plasmid DNA and plasmid DNA groups (*P* < 0.01).

### Effects of Bio-CS/plasmid DNA nanoparticles on cellular immunity in the orthotopic liver transplantation model

Following 10 days of treatment, the percentage of NK (CD3-CD49b+) and CTL (CD3+CD8+) cells in the spleen of the orthotopic liver transplantation model was significantly higher in the Bio-CS, plasmid DNA and Bio-CS/plasmid DNA groups compared with the corresponding percentages in the plasmid and control groups (*P* < 0.01) as shown in Figure 5C(a-b) and 5D(a-b). In addition, the percentage of NK and CTL cells was increased in the Bio-CS and plasmid DNA groups compared with the Bio-CS/plasmid DNA group (P <0.01). The highest percentage of these parameters was noted in the Bio-CS/plasmid DNA group (*P* < 0.01), whereas no significant differences between the plasmid and control groups were noted (*P* > 0.05). The percentages of Treg cells (CD4+CD25+Foxp3) were significantly lower in the Bio-CS, plasmid DNA and Bio-CS/plasmid DNA groups compared with those noted in the plasmid and control groups (Figure 5E(a-b), *P* < 0.01). The lowest percentage of Treg cells (CD4+CD25+Foxp3) was noted in the Bio-CS/plasmid DNA group (*P* < 0.01), whereas the difference was not significant between the plasmid and control groups (*P* > 0.05). The percentage of the CD3+, CD3+ CD4+ and CD3-CD19+ cell populations in the spleen of the orthotopic liver transplantation model was not significantly different among the different groups (Figure 5F, *P* > 0.05). The cytotoxicity of NK and CTL cells in the Bio-CS, plasmid DNA and Bio-CS/plasmid DNA groups was significantly higher compared with that noted in the plasmid and control groups (Figure 5G, *P* < 0.01). The cytotoxicity of NK and CTL cells was increased in the Bio-CS, plasmid DNA and Bio-CS/plasmid DNA groups (*P* < 0.01), whereas the highest cytotoxicity was noted in the Bio-CS/plasmid DNA group (*P* < 0.01). No significant differences were observed in the cytotoxicity of the plasmid and the control groups (*P* > 0.05).

## DISCUSSION

Nanoparticles demonstrate certain advantages for drug delivery and tissue targeting [16, 17]: (1) They can be effectively loaded with antitumor drugs, (2) they exhibit the target group on the particle surface and (3) they contain considerably small size that is estimated in the nanometer range. The target group of the receptors, should meet two criteria [18]: (1) Overexpression on the surface of tumor cells; and (2) low expression in normal cells. A variety of tumor cell target groups have been identified and grafted on the chitosan nanoparticles as potential drug carrier formulations [19, 20]. Hepatoma cells and several other cancer cells require a large amount of biotin, which is found in considerably higher concentrations compared with normal liver cells (39.6-fold higher) [14]. Liver cancer cells exert a strong adsorption effect on nanoparticles that have been modified with biotin. The adsorption of this preparation has been shown to increase with the biotin content [15]. In the present study, Bio-CS nanoparticles were successfully synthesized and validated using infrared and hydrogen^−1^ nuclear magnetic resonance (^1^H-NMR) spectroscopies. The degree of substitution of biotin was 35%. It was demonstrated that at a molar ratio of 1:1 of Bio-CS and plasmid DNA, optimal encapsulation rate was achieved, while the optimal CS and plasmid DNA nanoparticle ratio was 5:1. This finding indicated that Bio-CS was more efficient compared with the CS with regard to the encapsulation rate. Bio-CS was optimized for DNA encapsulation including additional side scions in the CS nanoparticle. This may lead to increased cellular uptake and gene transfection. However, the Bio-CS/plasmid DNA with a high ratio would conversely lead to decreased gene transfection. The number of free amino groups, which are positively charged at specific pH conditions, is one of the key parameters required for high DNA loading. The optimal pH-value of drug loading in the Bio-CS nanoparticle was 5.5. This value was different from that noted in the CS preparation, which indicated that CS was biotin-modified and that the positive charge of the nano-materials decreased, resulting in the optimal drug loading compared with CS alone. Transmission scanning electron microscopy indicated that the Bio-CS/plasmid DNA nanoparticles were spherical and exhibited a smooth surface, uniform size, homogeneous dispersion, and no adhesion and aggregation phenomena. The encapsulation efficiency reflected the quality of the nanoparticle preparation method, whereas the drug loading reflected the ability of the chitosan gene carrier. In the present study, the encapsulation and drug loading rates reached 85.7% and 35.4%, respectively. The diameter of nanoparticles was an additional parameter that affected the drug carrier system. The apertures of hepatic vascular endothelial cells and postcapillary venules were 2 nm and 6 nm, respectively, and the fenestrae size of the liver endothelial cells was approximately 106–175 nm. The diameters of the nanoparticles should be smaller than the aperture of liver endothelial cells in order for the liver targeting effect to take place. In the present study, the particle size of the Bio-CS/plasmid DNA nanoparticles was 82.9 nm, indicating that the nanoparticles could pass through the liver endothelial cells into the tumor tissues. A diameter of the nanomaterial/DNA complex of less than 100 nm is considered suitable for gene delivery [21]. As regards chitosan, the appropriate size and surface charge for transfection varies according to different studies. The latter particles are suitable for gene delivery when chitosan / DNA nanoparticles exhibit a diameter of the range of 50 to 200 nm and a zeta potential of the range of 15 to 37 mV 45-46 [22, 23]. In the current study, the radius of the nanoparticles was 82.9 nm and the zeta potential was +21.8 mV that indicated the suitability of the Bio-CS/plasmid DNA nanoparticles for gene delivery. It was demonstrated that Bio-CS/plasmid DNA nanoparticles exhibited a rapid and stable release period in simulated body fluid *in vitro*. The burst release of nanoparticles was observed prior to 15 h, since the plasmid that was localized on their surface was rapidly released into the simulated body fluid within a time period of 15 h. The material degraded gradually, and the plasmid was steadily released following 15 h.

Chitosan can bind with the negatively charged DNA via electrostatic interactions and forms a stable nanocomposite [24]. The protective ability of chitosan with regard to the DNA may be reduced with decreasing molecular weight. Chitosan may completely shield DNA at a molecular weight higher than 48 kDa [25], while it can reduce the existence of the free plasmid, protect DNA by inhibition of the degradation of nucleic acid enzymes in the blood and increase the DNA half-life [26, 27]. The current study indicated that the zeta potential of the Bio-CS/plasmid DNA nanoparticles that were synthesized using modified nanomaterials, was +21.8 mV. Gel retardation experiments demonstrated that the Bio-CS/plasmid DNA nanoparticles were inhibited to a major extent, indicating that the effective combination of the nanomaterials with the plasmid DNA exhibited a high encapsulation efficiency. The protection test of DNase *I* indicated that the Bio-CS nanomaterial could reduce the degradation of plasmid DNA. Following the digestion by DNase *I* for 30 min to 8 h, the electrophoresis strip was retained, which demonstrated that high concentrations of DNase *I* did not digest plasmid DNA in the nanoparticles. The concentration of nuclease used under physiological conditions was lower than the concentration of DNase *I* used under experimental conditions. Therefore, following digestion of plasma and Bio-CS/plasmid DNA nanoparticles for 8 h, a DNA strip could be displayed, whereas the plasmid DNA that was previously stored at 4 °C did not reveal a leakage from the gel holes during a 1 to 5 month period of storage. The DNA at 4 °C retained its functionality by storage in the TE buffer for 1 to 2 years. The Bio-CS nanomaterials exhibited a protective and stable effect on the DNA that was stored at 4 °C. It was suggested that the Bio-CS nanomaterials stored at 4 °C increased the protective effects on plasmid DNA and that they were considerably stable between 1 to 5 months, highlighting their immense potential as gene carriers.

The application of gene therapy for liver cancer exhibits certain difficulties, such as gene targeting, where an ideal gene carrier is required [28]. The present study indicated that the effect of endocytosis of the Bio-CS nanomaterials was enhanced compared with that of the CS nanomaterials in hepatocellular carcinoma cells. Gene transfection experiments further confirmed that Bio-CS nanomaterials exhibited higher gene transfer efficacy in hepatoma cells compared with CS nanomaterials, which indicated that Bio-CS nanomaterials exerted an apparent targeting effect on hepatocellular carcinoma cells. The main mechanism of action responsible for these effects was the endocytosis of nanoparticles in tumor cells. This process indicated that the cations on the surface of the nanoparticles were bound to glycoproteins and negatively charged phospholipids on the cell membrane, and were subsequently transferred to the cytoplasm. Hence, the cation number indicates a positive correlation with the gene transfer rate [29]. Concomitantly, biotin can couple with nanoparticles and facilitate their transfer to the cells in the presence of the targeting gene, which is considered a safe and effective targeting gene transfection method [30]. This occurs due to the binding effect of biotin with the corresponding biotin receptor on the cell surface. The dynamic imaging of Bio-GS nanoparticles was observed in the orthotopic liver transplantation model in mice. Biotin indicated the highest fluorescence intensity in liver cancer and normal liver cells compared with the CS nanoparticles. No difference was observed between CS and biotin preparations during targeting of the liver cancer cells. Bio-CS exhibited the highest fluorescence intensity in hepatocellular carcinoma cells and was more efficacious in liver cancer targeting compared to CS and biotin. These effects may be related to the characteristics of the nanomaterials: During intravenous administration of Bio-CS the nanoparticle was transported to the liver via the portal vein. Biotin ligands of the Bio-CS nanoparticle indicated a liver cancer targeting effect, whereas the concentration of nanomaterials was higher in the Bio-CS formulation, which demonstrated the ability of the nanoparticles to aggregate in the liver cancer tissues. Biotin alone was significantly less efficient in targeting the liver cancer cells compared with Bio-CS, which suggested that the spatial structure of CS was more conducive to the targeting of biotin to the liver cells. Competition and inhibition experiments on biotin demonstrated that in the presence of 30 mg/L of nanoparticles, the cellular uptake of biotin reached a saturation state and hence the increase in the concentration of nanoparticles could not further enhance the uptake of biotin from the liver cancer cells. Concomitantly, the addition of biotin to the culture medium could competitively inhibit the transfection of the Bio-CS/plasmid GFP nanoparticles on hepatocellular carcinoma cells that indicated the limited amount and saturation capacity of biotin receptors on the liver cell surface. The data further demonstrated that the combination effect of the biotin ligand and its receptor on the Bio-CS nanoparticles and hepatoma cells, respectively, could be applied by active transport in hepatoma cells that increased the affinity of Bio-CS nanoparticles for hepatoma cells significantly compared with the unmodified CS nanoparticles. In addition, the present study indicated that Bio-CS nanoparticles exhibited no significant cytotoxicity on hepatoma, liver and colon cancer cells at the concentration range of 0 to 0.64%. The toxicity noted in the aforementioned cell types was evident at a concentration of 2.56% of nanomaterials.

In order to verify whether the Bio-CS/plasmid DNA nanoparticles exhibited a targeted inhibitory influence on the orthotopic transplantation model of hepatocellular carcinoma cells, an orthotopic transplantation liver tumor model was established in mice, by injection of the Bio-CS/plasmid DNA nanoparticles to the tail vein of the mice. No significant differences among the plasmid DNA group, the empty plasmid, the Bio-CS, and the control groups were noted, suggesting that following degradation of the DNA by DNA enzymes, the plasmid DNA was transferred to the blood circulation. The Bio-CS/plasmid DNA and the CS/plasmid DNA groups that were protected by the nanomaterials exhibited a significantly increased inhibition of tumor growth in the orthotopic transplantation model. Furthermore, the survival time was significantly higher in the Bio-CS/plasmid DNA group compared with the nontargeted group of the CS/plasmid DNA. The number of mice that survived in the Bio-CS/plasmid DNA group was two and the survival rate was 22.22%, which indicated that the efficacy of the Bio-CS/plasmid DNA group was lower than that of the plasmid DNA group [17]. The exact etiology for this outcome requires further investigation, while the target materials may be improved in future experiments in order to enhance the therapeutic effect. The results indicated that Bio-CS nanomaterials are a promising novel gene delivery system, which can transfer DNA and achieve efficient targeting of the tumor cells. These processes improve the therapeutic effect and provide a new application for the gene vectors.

In the present study, Bio-CS significantly promoted the transfection of GM-CSF, IL-21 and Rae-1 in tumor cells, whereas the mRNA and protein expression levels of GM-CSF, IL-21 and Rae-1 in the spleen tissue decreased compared with those noted in the liver cancer tissues that included Bio-CS/plasmid DNA and plasmid DNA. This finding was consistent with the previous experiment conducted by our group suggesting that galactosylated chitosan can increase the gene transfection *in vivo* [3]. The Bio-CS / plasmid DNA increased significantly the gene and protein expression of GM-CSF, IL-1, as opposed to the Bio-CS group. The data were consistent with the study [31] that highlighted that Bio-CS is a suitable gene vector, which can increase gene transfection *in vivo*. Furthermore, Bio-CS / plasmid DNA has been shown to increase IFN-γ and IL-2 levels, whereas the ratio and cytotoxicity of NK and CTL cells was further reported to increase significantly, along with a concomitant reduction in the percentage of Treg cells. This finding is consistent with the previous study conducted by our group that indicated the capacity of the combination effect of GM-CSF, IL-21 and Rae-1 to enhance cellular immunity [7]. In the present study, the Bio-CS formulation was shown to promote cellular immune function. Several studies [32–34] have demonstrated that the skeleton of chitosan nanomaterials mimics the function of an immune activator, which can stimulate the immune function of the body, notably the cellular immunity. Qin and colleagues [35] demonstrated that chitosan exhibited a significant inhibition of tumor growth in tumor-bearing mice, due to potential activation of macrophages and dendritic cells [36–39] that in turn activate cytokine release by dendritic cells, increase the expression of the major histocompatibility complex II and activate T cells. The activated T cells can further release IL-2, IL-10, tumor necrosis factor-alpha and INF-γ, that in turn activate other immune cells in order to produce antibodies. Chitosan can activate the immune system by the following process [40]: The stimulation of the interferon gene pathway results in the induction of certain Interferons, which in turn mediate dendritic cell activation and induction of cellular immunity with a distinct Th1-associated profile. This indicates that a cationic polymer can induce the stimulation of interferon genes and the DNA sensing pathway in order to promote cellular immunity.

In conclusion, Bio-CS has been successfully synthesized and validated using infrared and ^1^H-NMR spectra. Bio-CS was more effective in promoting gene transfer through the cell membrane via endocytosis compared with CS nanomaterials, which is notably attributed to the combined effects of biotin and biotin receptors on nanomaterials and hepatocellular carcinoma cells, respectively. Bio-CS nanoparticles *in vivo* can mediate gene transfer and exert a significant inhibitory effect on the liver cancer cell model *in situ*, in the absence of apparent side effects on the cells. Taken together, the data suggest that Bio-CS nanomaterials enhance the transfection of plasmid DNA in hepatocellular carcinoma cells and activate the immune function

## MATERIALS AND METHODS

### Materials

Chitosan (CS) was from Zhejiang Aoxing Biotechnology Co., Ltd (Zhejiang, China), with 91% degree of deacetylation, a mean molecular weight of approximately 20 kDa and viscosity of 78 mPa.s. HCl (AR grade), *N*-hydroxysuccinimide (NHS), 1-ethyl-3-(3-dimethylaminopropyl) carbodiimide hydrochloride (EDC), Hoechst 33258 nuclear dye, plasmid green fluorescent protein (GFP) and RNase were obtained from Sigma-Aldrich (MO, USA). Biotin, calf serum, DNase I, RPMI 1640 powder, Cell Counting Kit-8 (CCK-8) and tetramethylethylenediamine (TEMED) were purchased from Beijing Jiakangyuan Pharmaceutical Co., Ltd (Beijing, China), Hangzhou Sijiqing Pharmaceutical Co., Ltd (Hangzhou, China), TaKaRa Biomedicals (Tokyo, Japan), Gibco/Invitrogen Inc (CA, USA), Dojindo Molecular Technologies Inc. (Shanghai, China)and Sinopharm Chemical Reagent Co., Ltd (Shanghai, China), respectively. The plasmid DNA (pGM-CSF-GFP-IRES-Rae-1-IL-21) was obtained as described before [7].

### Cell lines and experimental animals

Human hepatocellular carcinoma cells (SMMC-7721) and normal liver cells (LO2) were obtained from the Committee of Type Culture Collection of the Chinese Academy of Sciences (Shanghai, China). The human colon cancer cell line (SW480) was purchased from the American Type Culture Collection (VA, USA). The hepatocarcinoma 22 cell line (H22) was purchased from the Chinese Center for Type Culture Collection (CCTCC, Wuhan, China).

Female BALB/c mice that were 6-7 weeks old were obtained from the Science Department of Experimental Animals of Shanghai University of Traditional Chinese Medicine in China. All animals were housed in SPF room of animal house in Tongji University. All animals were treated following the protocol approved by the Institutional Animal Care and Use Committee at the Shanghai Institute of Materia Medica, Chinese Academy of Sciences.

### Preparation of Bio-CS

Biotin was first dissolved in NHS, and dicyclohexylcarbodiimide was added following dissolution of biotin. Following 16 h, crude bio-NHS was synthesized, and the pure bio-NHS that was obtained following the by-products of crude bio-NHS, such as dicyclohexylurea, was removed and further purified. Subsequently, Bio-NHS solution (6g/L) of an approximate volume of 4 mL was added dropwise in a 20 mL solution of CS material (30%) and the resulting solution was incubated at room temperature under magnetic stirring (78-1, Changzhou Aohua Instrument Co., Ltd, Changzhou, China) for 72 h. The resulting mixture was subjected to dialysis with distilled water for 4 days, and the dialysate was changed every 24 h. The galactosylated chitosan-graft biotin (Bio-CS) materials were collected following dialysis and freeze-drying. The average molecular weight (Mw) was estimated as the mean value of the weight distribution of the molecular sizes that were defined by the formula Mw=Σw_i_M_i_/Σw_i_. where M_i_ and w_i_ were the molar and the total mass of the molecular species.

### Fourier transformation infrared spectroscopy

CS and Bio-CS powder were mixed in a potassium bromide pellet. The composites were analyzed using Fourier-transformed infrared spectroscopy (FT-IR) (NEXUS, Nicolet/Natus Medical Incorporated, CA, USA) in the range of 4,000 to 400 cm^–1^.

### ^1^H-NMR analysis

In order to verify the structure of the CS and Bio-CS formulations, the samples were dissolved in a solution of deuterium chloride (12%) and D_2_O. ^1^H-NMR spectra were recorded using a Varian NMR System 600 machine (Varian, Inc./Agilent Technologies, CA, USA) at a resonance frequency of 600 MHz. Tetramethylsilane was used as a reference compound.

### Biotin content estimation on the surface of the nanoparticles

Biotin quantitative detection kit (cat. No. BDK-2000, Vector) was used to determine the biotin content on the surface of the nanoparticles (200mg biotin). The procedure was briefly described as follows: A total of 50 μL of each standard and/or sample was added to each well, and 50 μL of horseradish peroxidase that was labeled with avidin working solution was subsequently added. The resulting mixture was incubated at 37 ° C for 30 min and washed 5 times. A total of 90 μL of substrate solution was added in each well, and the samples were incubated at 37 ° C for 15 min. A total of 50 μL of stop solution was added at the end of the experiment. All samples were measured at a wavelength of 450 nm, and a standard curve was prepared by curve expert 1.3 software in order to determine the corresponding concentration based on the OD values.

### Synthesis of bio-CS/plasmid DNA nanoparticles

The Bio-CS (52 kDa) and/or CS materials and plasmid DNA, respectively, were added in the centrifuge tube and were incubated in a water bath (pH from 5.1 to 5.7) at a temperature of 45 to 50 °C for 5 to 10 min. The Bio-CS/ plasmid DNA was mixed at a molar ratio range of 1:0.25, 1:0.5, 1:1, 1:1.5, and 1:2, while the CS/ plasmid DNA was mixed at a molar ratio range of 3:1, 4:1, 5:1, 6:1 and 7:1 in a solution, using a vortex oscillator at 2,000 revolutions per minute (rpm) for 30 s. The resulting sample was kept at room temperature for 30 min. The suspensions of nanoparticles were centrifuged at 4,000 rpm, the centrifugal supernatant was collected and the residues were washed in a large volume of deionized water, freeze-dried, and stored for further analysis. Subsequently, a concentration range of nanoparticles according to each experiment was prepared. The zeta potential and average particle size of nanoparticles were detected using Zetasizer-3000HS (Malvern Instruments, Malvern, UK), while their shape was determined using TECNA10 transmission electron microscope (TEM, Philips Company, Netherlands). The encapsulation efficiency was determined using ultraviolet (UV)-spectrophotometry (Beckman; DU series 650, NC, USA) in order to detect the concentration of DNA in the collected supernatant, whereas the formulas for the calculation of the encapsulation efficiency and the drug loading were as follows:

$$\text{Drug loading}(\%)=\frac{\text{DNA in nanoparticles}}{\text{Nanoparticles mass}}\times 100\%$$

### *In vitro* release experiment

Bio-CS/Plasmid DNA nanoparticles (10 mg) were mixed with 1 mL of simulated body fluid (SBF; 0.11 mol/L of sodium chloride, 0.005 mol/L of potassium chloride, 0.0025 mol/L of calcium chloride, 0.0012 mol/L of monopotassium phosphate, 0.0024 mol/L of magnesium sulfate, 0.0017 mol/L of sodium bicarbonate and 0.0013 mol/L of disodium hydrogen phosphate at pH 7.4) solution in dialysis bags and incubated at 37 °C using a shaker at a constant speed of 60 rpm. A total of 10 μL of the supernatant was collected following centrifugation every hour. The concentration of the plasmid DNA that was released in the centrifugal supernatant at different time points was measured at 260 nm using UV-spectrophotometry. The characteristics of the plasmid DNA released from the Bio-CS/plasmid DNA nanoparticles *in vitro* were subsequently observed. Each experiment was carried out in triplicate. The cumulative release percentage formula was as follows:

$$\text{Cumulative release percentage}(\%)=\frac{\text{Release of plasmid DNA in the medium}}{\text{Total plasmid DNA of Bio}-\text{CS}/\text{plasmid DNA nanoparticles}}\times 100\%$$

### Effect of bio-CS on plasmid DNA

In order to detect the protective effects of Bio-CS for the plasmid DNA, the Bio-CS/plasmid DNA nanoparticles were digested with DNase I and observed by gel electrophoresis. Electrophoresis was carried out with Bio-CS and plasmid DNA as control groups, and the plasmid DNA was digested with DNase I at 37 °C for 1 h. The Bio-CS/plasmid DNA nanoparticles were digested with DNase I at 37 °C for 30 min, 1 h, 2 h, and 8 h, and with plasma at 37 °C for 8 h. The Bio-CS/plasmid DNA nanoparticles were stored at 4 °C for a period of 1 to 5 months. The DNA analysis was conducted by 1% agarose gel electrophoresis and the effects were observed using a UV transilluminator (Kodak Digital Science, NY, USA).

### Cell staining and confocal imaging

Two types of cell lines namely, SMMC-7721 and LO2 were seeded in 6-well plates (each well was placed in a clean round cover slip) and incubated for 24 h. Fresh medium was added, which contained nanoparticles with different fluorothioisocynate (FITC) markers (CS and Bio-CS nanoparticles), and the cells were incubated for 4 h at 37 °C. The cells were fixed with 4% paraformaldehyde at room temperature for 20 min. The nuclei were stained with Hoechst 33258 and washed three times with 0.01 mL of PBS. The cover slips were removed with tweezers and were put on a glass slide, mounted with glycerol buffer solution and observed using a confocal laser scanning microscope (Olympus FV-1000, Tokyo, Japan) for fluorescence imaging. The excitation wavelength of the fluorescent nanoparticles was 488 nm and the emission wavelength 405 nm. The images were photographed using NIS-Elements imaging software.

### Cell transfection

SMMC-7721 and LO2 cells were grown in logarithmic phase and counted following digestion and centrifugation. The cells were seeded in 24-well plates at a density of 5×10^5^ cells per well and incubated overnight in a humidified incubator containing 5% CO2 at 37 °C. The cells were further treated with different concentrations of Bio-CS/plasmid GFP and CS/plasmid GFP nanoparticles (5, 10, 15, 20, 25, 30, 35, 40, 45, 50, 55, and 60 mg/L) for 48 h. Subsequently, the cultured cells were observed under a fluorescent microscope (Olympus, Tokyo, Japan) and the transfection efficiency was determined by flow cytometry (Beckman-Coulter Inc., CA, USA).

### Biotin inhibition test

SMMC-7721 cells were seeded in 96-well plates and incubated for 24 h. The cells were treated with different concentrations of biotin (0, 0.2, 0.4, 0.6, 0.8, and 1.0 mM) for 4 h. Following removal of the supernatant and two rounds of washing by cold PBS, 100 μL of Bio-CS/plasmid GFP and CS/plasmid GFP nanoparticles (4 μg plasmid GFP) were added to each well and incubated for 4 h. The cells were harvested, washed and lysed. The fluorescence intensity was measured from five wells at different concentration levels of biotin that corresponded to each type of nanoparticle used.

### Cytotoxicity of bio-CS nanomaterials

The cytotoxicity of the Bio-CS nanomaterials was determined by the CCK-8 assay. SMMC-7721, SW480 and LO2 cells were cultured in RPMI-1640 medium supplemented with 10% fetal calf serum in the presence of 5% CO_2_ at 37 °C. A total of 200 μL containing different concentrations of Bio-CS nanoparticles (0, 0.01%, 0.04%, 0.16%, and 0.64%, 2.56%) were dissolved in RPMI-1640 and added to each well. SMMC-7721, SW480 and LO2 cells, in the absence of nanoparticles, were added to the negative control groups. RPMI-1640 medium was solely used for the blank control group. Following incubation for 3 days, 100 μL of CCK-8 was added to each well and incubated for 1 h. The absorbance was measured using a Bio-Rad automatic microplate reader (Bio-Rad, CA, USA) at 450 nm. All measurements were conducted in triplicate.

$$\text{Cell proliferation ratio}(\%)=\frac{{\text{OD}}_{\text{Treated group}}-{\text{OD}}_{\text{Blank group}}}{{\text{OD}}_{\text{Nagative group}}-{\text{OD}}_{\text{Blank group}}}\times 100\%$$

### Orthotopic transplantation liver cancer model

H22 cells were injected to mice in order to establish the subcutaneous liver cancer mouse model. The mice were sacrificed in order to harvest the tumor tissues following 30 min of excessive administration of anesthetic. Fresh fast-growing tumor tissues were selected and were collected in a tumor cell suspension at a density of 6 × 10^7^/mL. Recipient mice were anesthetized with 20% urethane, and the suspension (50 μL) was injected to the liver left lobe capsule. In the absence of leakage, the abdomen was closed following 2 min and the orthotropic liver cancer mouse model was established successfully.

### Targeting detection of bio-GC nanoparticles *in vivo*

Bio-CS with 0.5 g of CS and/or biotin was dissolved in 100 mL of 1% (v/v) acetic acid, stirred for 30 min and the pH of the solution was adjusted to 7.5 with small additions of 1 mol/L of NaOH solution. A total of 1 mg of Rhodamine B isothiocyanate (RBITC) was dissolved in l mL of DMSO in order to yield a 1 g/L solution of RBITC. The RBITC solution (1 mL) was added dropwise in the Bio-CS solution at 40 °C in a water bath under electromagnetic stirring. The reaction was conducted for 1 h and the samples were allowed to react overnight at room temperature. The RBITC-Bio-CS solution was subjected to dialysis with distilled water for 3 days in order to remove the non-bound RBITC, whereas RBITC-Bio-CS materials were collected following dialysis and they were freeze-dried. The samples were stored at 4 °C.

The orthotopic transplantation liver cancer model was established following day 5 in order to ensure the consistency of the fluorescence signal intensity of the modified nanoparticles. The RBITC-Bio-CS, RBITC-CS, and RBITC-biotin (200 μL) formulations were injected to the tail vein at a concentration of 2 g/L. The dynamic distribution of CS, biotin and Bio-CS in the mice was detected using a small-animal *in vivo* imaging system (CRi, Maestro, CA, USA) at the time points of 2, 4, 8, 12 and 24 h. A total of 3 mice from each group were sacrificed at each time point following 30 min of overdose treatment with anesthetic, and the fluorescence intensity that corresponded to the liver, kidney, spleen, and brain tissues was detected. The mice were sacrificed at 24 h. The fluorescence photon numbers of normal liver and liver cancer regions were detected using the CRi Maestro imaging detection system, and the hepatic cancer (C) and normal liver (L) fluorescence ratios were calculated.

### Bio-CS/plasmid DNA nanoparticles in the orthotropic liver cancer mouse model

Hepatic tumors were grown in mice to a diameter range of 4 to 6 mm. The mice with tumors were randomly divided into 6 groups namely, control, Bio-CS, plasmid, plasmid DNA, CS/plasmid DNA, and Bio-CS/ plasmid DNA. The mice of the control, Bio-CS, Bio-CS nanomaterial plasmid, plasmid DNA, CS/plasmid DNA and Bio-CS/plasmid DNA groups received 200 μL of saline, Bio-CS, Bio-CS nanomaterial and plasmid DNA and CS/plasmid DNA (containing 100 μg plasmid DNA), respectively by intravenous injection. The drugs were administered continuously for 5 days initially from day 6 following establishment of the model. Following 10 days of treatment, the mice were sacrificed and the tumor and spleen tissues were assessed by RT-PCR and Western blotting assays in order to determine the expression of GM-CSF, IL-21 and Rae-1. A total of 9 mice in each group were used for survival analysis.

### ELISA assays

Mouse serum specimens were isolated from 3 mouse models following treatment with Bio-CS/plasmid DNA nanoparticles at day 10 in each group and kept at -20 °C for the determination of IL-2 and INF-γ content. The serum specimens were thawed at 37 °C in an incubator, followed by dilution with double distilled water to   μL for ELISA (Santa Cruz Biotechnology, CA, USA) detection. Furthermore, the IL-2 and INF-γ standards were diluted to 8,000 μg L^−1^. The reactions were carried out in a 96-well plate (Sigma-Aldrich) and each well contained 150 μL of diluted specimens and 50 μL of standards. The samples were oscillated evenly and incubated for 2 h at room temperature. The liquid in the wells was removed and the wells were washed with 400 μL of detergent 4 times, followed by the addition of horseradish peroxidase for the detection of IL-21 and INF-γ (200 μL). Subsequently, the samples were oscillated evenly and incubated for an additional 2 h at room temperature. The liquid was removed from the samples and an additional wash with 400 μL of detergent was conducted for four times. Equal amounts of developer A and B were evenly mixed and incubated with 200 μL streptavidin-HRP conjugated anti-mouse antibody at room temperature in the dark for 30 min. A total of 50 μL stop buffer was added to terminate the enzymatic reaction. The specimens were immediately analyzed for optical density detection at a wavelength of 450 nm. The samples and the standards were run in triplicate and the sensitivity of the assay was approximately 0.1 units/mL for both IL-2 and INF-γ.

### Reverse transcription-polymerase chain reaction (RT-PCR)

Total RNA was extracted using Trizol reagent (TaKaRa Biotechnology Co., Ltd., Tokyo, Japan). The sequences of the PCR sense and anti-sense primers for the genes investigated were as follows:

GM-CSF: forward, 5’-GAGGTACCAGATCACCGGCGAAGGA-3’; and reverse, 5’-TATAAGCTTGCTTCCTCATTTTTGGCCTGG-3’

IL-21: forward, 5’-CCGCTAGCCTGGAGACTCAGTTCTG-3’; and reverse, 5’-CCCAAGCTTCTAGGAGAGATGCTGATG-3’

glyceraldehyde-3-phosphatede hydrogenase (GAPDH) gene are forward, 5’-50-ACCGCAAA GACTGTGGATGC-3’; and reverse, 5’-TGAGCTTGAC AAAGTGGTCG-3’.

The primers were synthesized by the Shenneng Company of Gene and Technology in Shanghai, China. Reverse transcription was conducted using 1 μL of RNA, 0.5 μL of AMV reverse transcriptase, 2.5 μL cDNA as a template for PCR amplification, 0.1 μL polymerase Ex Taq HS, 0.1 μL of sense primer and 0.1 μL of antisense primer. The reaction conditions were the following: initial denaturation for 2 min at 94 °C, 35 cycles of 3 steps namely, denaturation for 40 s at 94 °C, annealing for 40 s at 50-65 °C and extension for 1 min at 72 °C and a final extension at 72 °C for 5 min. The PCR products were kept frozen at -20 °C. The GAPDH gene was used as an internal housekeeping control and was amplified using the aforementioned conditions. The mRNA expression levels of GM-CSF, IL-21, Rae-1 and GAPDH were detected by DNA gel (2% agarose) electrophoresis of the cDNA corresponding to the aforementioned gene transcripts. Following electrophoresis, the gel was placed in ethidium bromide (EB) staining solution for 5 min.

### Western blot analysis

Primary antibodies used were anti-GM-CSF (Santa Cruz Biotechnology, Santa Cruz, CA), anti-IL21 (Gen Way Biotech, San Diego, CA), and anti-GAPDH (Sigma-Aldrich, USA). The proteins were electrophoresed initially at 80 V and subsequently at 120 V for 1.5 h. Polyvinylidene fluoride fibre (PVDF) membrane was immersed in methanol for 5 min and transferred to buffer solution (pH 8.3, 25 mmol L^−1^ Tris-HCl, 192 mmol L^−1^ glycine, and 20% methanol) for 10 min. The proteins were transferred on PVDF membranes via electrophoresis at 100 V for 70 min, and subsequently blocked with 5% bovine serum albumin/PBS by overnight incubation at 4 °C. The following morning, the membrane was incubated with primary antibodies (1:2,000 dilutions) for 60 min at room temperature and was subsequently rinsed in 0.05% PBS-Tween 20 3 times for 10 min each. The membrane-bound protein-primary antibody complexes were incubated with secondary antibodies (1:8,000 dilutions) for 3 h. The membranes were rinsed in 0.05% PBS-Tween 20 3 times for 10 min each and the detection was conducted by ECL reagents. Equal parts of solutions A and B in the ECL kit were mixed and the resulting solution was incubated with the membrane for 1 min. The proteins were developed and photographed by a gel imaging system.

### Flow cytometry

The spleens that were harvested from the mice were carefully filtered against mesh screens to obtain single-cell suspensions, which were incubated in RPMI 1640. Cells were seeded into a 96-well plate, followed by centrifugation at 300 *g* for 5 min. Subsequently, cell pellets were retained and washed 2 to 3 times with 200 μL of PBS. The cells were mixed with a blocking agent and incubated under gentle shaking at 4 °C for 60 min, followed by 2 to 3 washes with 200 μL of PBS. Primary antibodies (anti-CD3, anti-CD4, anti-CD8, anti-CD25, anti-CD19, anti-CD49b, anti-H60, anti-NKG2D and anti-Foxp3 antibodies) were added to their respective wells, incubated at 4 °C under gentle shaking for 60 min, and washed 2 to 3 times with 200 μL of PBS. Subsequently, the cells were resuspended in 200 μL of ice-cold PBS and kept in the dark for determination of the total numbers of T cells. Specifically the following subsets of T cells were determined: CD3+, Th cells of the CD3^+^ CD4^+^ phenotype, CTL cells of the CD3^+^ CD8^+^ phenotype, B cells of the CD3^−^ CD19^+^ phenotype, NK cells of the CD3^−^ CD49b^+^ phenotype, and Treg cells expressing CD4^+^ CD25^+^ Foxp3^+^. The analysis was carried out by flow cytometry (BD Biosciences, Franklin Lake, NJ, USA).

### MTT detection of CTL and NK activity

Using the random number method, 3 mice models were detected by MTT in each group. Spleen cells (NK and CTL cells) were selected by flow cytometry. The NK and CTL cell populations were observed by fluorescence microscopy and their concentrations were adjusted to 1×10^6^/mL. YAC-1 and H22 were used as target cells for NK and CTL reactivity, respectively. The cells were incubated in RPMI 1640 containing 10% of fetal bovine serum at 37 °C in 5% of CO_2_ for 48 h. Cell density was adjusted to 1×10^5^/mL. A total of 3 mice were randomly selected in each group for the detection of CTL and NK cell activity. A total of 100 μL of spleen and target cells (at an effector:target cell ratio of 10:1) were added to a 96-well staining plate. Following 18 h of incubation, MTT (20 μL) was added to each well and incubated for 4 h. Following centrifugation, the supernatant was removed. Dimethyl sulfoxide (DMSO) (200μL) was added to the plate and agitated for 20 min. Absorbance was determined at a wavelength of 570 nm (A_570_). The activity of CTL and NK cells was calculated as follows:

$$\text{Activity}=\frac{1-({\text{A}}_{570}\text{of effector}-\text{target cells}-A_{570}\text{of effector cells})}{A_{570}\text{of target cells}}\times 100\%$$

### Statistical analysis

All data were expressed as mean ± standard deviation. One-way analysis of variance (ANOVA) and the least significance difference (LSD) test were used to analyze between-group data. A P-value of less than 0.05 (P<0.05) was considered statistically significant.

## Abbreviations

Bio-CS: biotinylated chitosan
Bio-GC: biotin-modified galactosylated chitosan nanoparticles
FT-IR: fourier transformed infrared spectroscopy
^1^H-NMR: hydrogen-1 nuclear magnetic resonance
GFP: green fluorescent protein
CCTCC: China Center for Type Culture Collection
NHS: N-hydroxysuccinamide
TEM: transmission electron microscope
FITC: fluorothioisocynate
RPMI: Roosevelt Park Memorial Institute
RBITC: Rhodamine B isothiocyanate
ANOVA: analysis of variance
CCK-8: Cell Counting Kit-8
INF-γ: Interferon-γ
IL-2: Interleukin-2
ELISA: enzyme linked immunosorbent assay
GM-CSF: granulocyte macrophage-colony stimulating factor
IL-21: interleukin-21
Rae-1: retinoic acid early transcription factor-1
Th: helper T
CTL: cytotoxic lymphocyte
NK: natural killer cell
Treg: regulatory cells
MTT: methylthiazolyldiphenyl-tetrazolium bromide
